# The effects of 18-h fasting with low-carbohydrate diet preparation on suppressed physiological myocardial ^18^F-fluorodeoxyglucose (FDG) uptake and possible minimal effects of unfractionated heparin use in patients with suspected cardiac involvement sarcoidosis

**DOI:** 10.1007/s12350-015-0226-0

**Published:** 2015-08-05

**Authors:** Osamu Manabe, Keiichiro Yoshinaga, Hiroshi Ohira, Atsuro Masuda, Takahiro Sato, Ichizo Tsujino, Asuka Yamada, Noriko Oyama-Manabe, Kenji Hirata, Masaharu Nishimura, Nagara Tamaki

**Affiliations:** Department of Nuclear Medicine, Hokkaido University Graduate School of Medicine, Sapporo, Japan; First Department of Medicine, Hokkaido University Hospital, Sapporo, Japan; Diagnostic and Interventional Radiology, Hokkaido University Hospital, Sapporo, Japan; Molecular Imaging Research Center, National Institute of Radiological Science, 4-9-1 Anage, Inage-Ku, Chiba 263-8555 Japan

**Keywords:** Cardiac sarcoidosis, ^18^F-fluorodeoxyglucose, positron emission tomography, long fasting, free fatty acid

## Abstract

**Background:**

^18^F-fluorodeoxyglucose (FDG) PET plays an important role in the detection of cardiac involvement sarcoidosis (CS). However, diffuse left ventricle (LV) wall uptake sometimes makes it difficult to distinguish between positive uptake and physiological uptake. The aims of this study were to evaluate the effects of 18-h fasting with low-carbohydrate diet (LCD) vs a minimum of 6-h fasting preparations on diffuse LV FDG uptake and free fatty acid (FFA) levels in patients with suspected CS.

**Methods:**

Eighty-two patients with suspected CS were divided into 2 preparation protocols: one with a minimum 6-h fast without LCD preparation (group A, n = 58) and the other with a minimum 18-h fast with LCD preparation (group B, n = 24). All patients also received intravenous unfractionated heparin (UFH; 50 IU/kg) before the injection of FDG.

**Results:**

Group A showed a higher percentage of diffuse LV uptake than did group B (27.6 vs 0.0%, *P* = .0041). Group B showed higher FFA levels (1159.1  ±  393.0, 650.5  ±  310.9 μEq/L, *P* < .0001) than did group A. Patients with diffuse LV uptake (n = 16) showed lower FFA levels than did other patients (n = 66) (432.1  ±  296.1, 888.4  ±  381.4 μEq/L, *P* < .0001). UFH administration significantly increased FFAs in both groups, even in the patients with diffuse LV FDG uptake.

**Conclusions:**

The 18-h fast with LCD preparation significantly reduced diffuse LV uptake and increased FFA levels. In particular, the FFA level was significantly lower in patients with LV diffuse uptake than in patients without LV diffuse uptake. Acutely increasing plasma FFA through the use of UFH may not have a significant role in reducing physiological LV FDG uptake.

## Introduction

^18^F-fluorodeoxyglucose (FDG) positron emission tomography (PET) plays an important role in the detection of active inflammatory lesions in cardiac involvement sarcoidosis (CS).^1^-^4^ Recently, the Heart Rhythm Society (HRS) issued a consensus statement on the diagnosis and management of arrhythmias associated with CS.^5^ In this statement, FDG PET was recommended for advanced cardiac imaging in CS diagnosis. A FDG PET preparation protocol and image interpretation should therefore be improved and standardized. The Japanese Society of Nuclear Cardiology (JSNC) has also issued FDG PET imaging guidelines for diagnosis CS.^1^ The positive uptake pattern is defined using visual assessment in HRS and JSNC guidelines. By adding a quantitative parameter over the standard visual analysis, FDG PET may improve the ability to diagnose patients with CS. For the evaluation of FDG uptake, volume-based parameters were proposed to estimate disease progression in the assessment of malignant tumors.^6^,^7^ Ahmadian et al also applied FDG volume measurement for patients with cardiac sarcoidosis.^8^

Sarcoidosis is a systemic granulomatous disease with unknown etiology.^9^ Manifestation of sarcoidosis varies from an asymptomatic state to a progressive disease, and CS has the greatest life-threatening potential in sarcoidosis patients.^10^ Therefore, the precise diagnosis of CS is important to sarcoidosis patients. The heart uses a variety of energy sources such as free fatty acids (FFA), glucose, lactate, and ketone bodies.^11^,^12^ FDG is an analog of glucose. Theoretically, glucose use during a fasting state is limited but diffuse left ventricle (LV) wall FDG uptake is sometimes reported even under fasting conditions.^2^,^13^ In addition, FDG uptake in the entire LV wall is generally considered to be physiologically normal.^1^ This physiological uptake makes it difficult to distinguish between active inflammation and physiological distribution.^1^,^14^ Myocardial FDG uptake is known to be correlated inversely with serum FFA concentration but not with serum insulin concentration.^15^-^17^ Several recent studies have focused on reducing physiological FDG uptake through such measures as performing extended fasting, following a particular regime (a low-carbohydrate diet or a high-fat diet), or receiving unfractionated heparin (UFH), and they have examined the usefulness of provoking a shift from glucose metabolism to FFA metabolism.^1^,^2^,^18^-^20^ However, there have been limited data to evaluate the association between certain details of FFA level and diffuse LV FDG uptake under a longer fasting period and LCD in patients with suspected CS. In addition, the effect of UFH in reducing diffuse LV FDG uptake has not been established. The aims of this study were to examine the effect of the preparations on diffuse LV FDG uptake and to evaluate the association between diffuse LV FDG uptake and FFA levels before and after UFH injection. We also tried to evaluate the cardiac FDG uptake volume and compare that evaluation to the visual assessment.

## Materials and Methods

### Subjects and Study Design

This study protocol was approved by the ethics committee of the Hokkaido University Graduate School of Medicine. Written informed consent was obtained prior to the study. Patients with suspected CS who underwent FDG PET or PET/CT from February 2002 to June 2013 were prospectively included in the current study. Patients were excluded if they lacked blood samples or the latter were insufficient for the purpose of measurements. Patients with a history of coronary artery disease (CAD) were also excluded from this study. We also excluded patients with any other known heart disease such as myocarditis, valvular heart disease, or other cardiomyopathies as identified by echocardiography. Out of 95 patients, 13 were excluded because they lacked blood samples or a large enough blood sample for detailed measurements (n = 12), or because they had hypoglycemia before the FDG PET/CT scan (n = 1).

### Patient Group

The final study population comprised 82 patients (22 men and 60 women), ranging in age from 22 to 76 years (54.1 ± 15.8 years). Patients were sequentially divided into 2 groups based on their FDG preparation protocol. Between February 2002 and April 2010, 58 patients underwent at least 6 h’ fasting without LCD (group A). After May 2010, 24 patients underwent over 18 h’ fasting with LCD (group B). The patients in group B ate a LCD with less than 5 g of carbohydrate per meal along with a boiled egg, tofu with bonnet flakes, and grilled chicken breast with stir-fried vegetables.^2^ Diabetic medications were skipped beginning in the morning. Long-acting insulin was skipped beginning the previous night. Patients were not randomly divided between the 2 protocols. Rather, they were placed into one of the two protocols according to the timeframe of the study. Group A (the 6 h fast group) was formed prior to May 2010; group B (the 18 h fast group) was formed after May 2010. In group A, we further divided the patients into two groups based on FDG findings: positive for diffuse LV FDG uptake (group A1) and negative for diffuse LV FDG uptake (group A2).

### Heparin Injection and Blood Sampling

**Figure 1:**
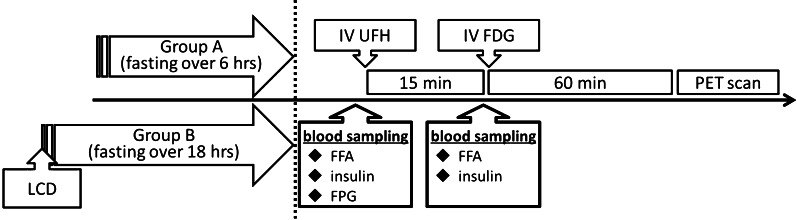
FDG PET data acquisition protocol. Group A (n = 58): minimum 6 h’ fasting before FDG PET studies. Group B (n = 24): minimum 18 h’ fasting with low-carbohydrate diet. Both groups had unfractionated heparin (UFH) administration 15 min prior to FDG injection. Scans were performed at 60 min after the administration of FDG. Blood samples were obtained before and 15 min after UFH administration to measure immunoreactive insulin (IRI) and free fatty acids (FFA). Fasting plasma glucose (FPG) level was measured before UFH injection

Prior to FDG PET imaging, we checked for contraindication of heparin use such as active bleeding, bleeding risk, or a history of heparin-induced thrombocytopenia. None of the patients in this study population had contraindication for heparin use. All patients received intravenous unfractionated heparin (UFH; 50 IU/kg, Mochida, Tokyo) 15 min prior to FDG injection.^14^,^21^ Before and 15 min after UFH administration, blood samples were obtained to measure immunoreactive insulin (IRI) and FFA. Fasting plasma glucose (FPG) level was also measured before FDG injection (Figure 1).

### FDG PET or PET/CT Imaging

PET data acquisition was performed using a Siemens ECAT Exact 47 or Siemens ECAT Exact HR + scanner (Siemens/CTI, Knoxville, TN, USA) (n = 50). PET/CT imaging was performed using a Biograph 64 TruePoint with TrueV (Siemens Japan, Tokyo) (n = 32). Transmission scanning for PET or a low-dose CT for PET/CT was performed for attenuation correction. Sixty minutes after FDG administration, a static FDG scan was performed.^1^ All scans were performed using whole-body imaging.

### FDG PET or PET/CT Imaging Analysis

At first, LV FDG myocardial uptake was evaluated visually. Definite FDG uptake in the entire LV wall higher than liver uptake was defined as diffuse LV FDG uptake based on JSNC guidelines.^1^ Two nuclear medicine physicians blinded to clinical and other test data independently evaluated the LV myocardial FDG images.

We investigated the glucose metabolic status of the cardiac lesions using maximal standard uptake value (SUVmax) and cardiac metabolic volume (CMV). SUVmax was measured using a volume of interest inserted on the fused axial image encompassing the entire heart, and reviewed to ensure that adjacent non-cardiac FDG-avid structures were excluded. To calculate the SUVmax of the blood pool, a region of interest (ROI) was drawn around the left atrial lumen.^22^ For the volume-based assessment, the whole process was done using dedicated software.^7^ The algorithm required human interaction in the first step to put a 150-mm spherical volume of interest (VOI) in the right lobe of the liver. The subsequent processes were automated. The within-VOI mean and standard deviation (SD) were used to determine a threshold value as follows: threshold = mean + 3 × SD.^23^ An experienced nuclear medicine physician thoroughly reviewed the PET images and selected the FDG-avid areas in the LV region. In cases in which LV uptake and uptake in adjacent structures (such as lymph node) overlapped, the overlap was carefully adjusted for manually. The nuclear physician was blinded to the clinical information and outcomes to avoid possible bias in software operation. Two patients were excluded because of multiple liver uptakes due to sarcoidosis.

### Statistical Analyses

Data are expressed as mean ± standard deviation (SD), and a *P* value of less than .05 is considered statistically significant. For intra-group comparisons, Wilcoxon signed-rank tests were applied to compare differences in the blood samples between the patients in the 6-h fasting group and those in the 18-h fasting group. Fisher’s exact test was used to compare discrete data as appropriate. Statistical calculations were carried out using statistical software (JMP version 10, SAS Institute, Cary, NC, USA).

## Results

### Patient Characteristics

Ninety-four patients did not have any issues under long fasting preparation and heparin use. One patient with over 18 h’ fasting experienced hypoglycemia before FDG administration and this patient did not undergo FDG PET study.

Between the two groups, there were no significant differences in baseline characteristics including sex, age, frequency of the patients who met the Japanese Ministry of Health and Welfare criteria (JMHW),^24^ left ventricular ejection fraction (LVEF), rate of focal uptake, and steroid use (Table 1).

**Table 1:** Patient characteristics

	Group A (n = 58)	Group B (n = 24)	*P* value
Sex (male)	16 (27.6%)	6 (25.0%)	1.00
Age (years old)	53.0 ± 16.2	56.7 ± 14.6	.42
Meets JMHW criteria	18 (31.0%)	12 (50.0%)	.11
Fasting time	Not available	20.6 ± 1.3 h	
Rate of focal uptake	41.4 (%)	54.2 (%)	.29
Diabetes	2 (3.4%)	2 (8.3%)	.58
LVEF	64.8 ± 12.9	66.7 ± 11.8	.56
Steroid therapy before PET scan	6 (10.3%)	3 (12.5%)	.72

Group A: patients with minimum 6 h fasting; Group B: patients with minimum 18 h fasting and LCD preparation

*JMHW*, Japanese Ministry of Health and Welfare; *LVEF*, left ventricular ejection fraction estimated by echography

### FDG PET or PET/CT Findings

**Figure 2:**
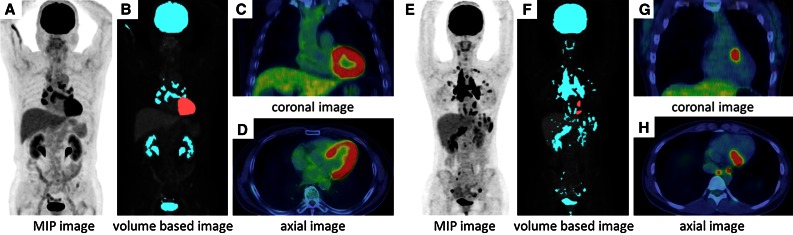
Representative cases. Figure (**A-D)** shows a 73-year-old man with diffuse LV FDG uptake, who was instructed to fast for a minimum of 6 h and whose FPG and FFA levels were 95 mg/dL and 464 μEq/L at baseline. Cardiac metabolic volume was estimated as 166.4 mL. There are multiple abnormal uptakes in mediastinal and hilar lymph nodes. Figure (**E**-**H)** shows a 25-year-old woman without diffuse LV FDG uptake, who had over 18 h’ fasting with a low-carbohydrate diet. Her FPG and FFA levels were 76 mg/dL and 1924 μEq/L at baseline. FDG PET/CT shows focal basal anterior wall uptake. Cardiac metabolic volume was estimated to be 8.3 mL. There are multiple lung uptakes and multiple lymph node uptakes in supraclavicular, mediastinal, hilar, and abdominal regions. MIP = maximum intensity projection

**Figure 3:**
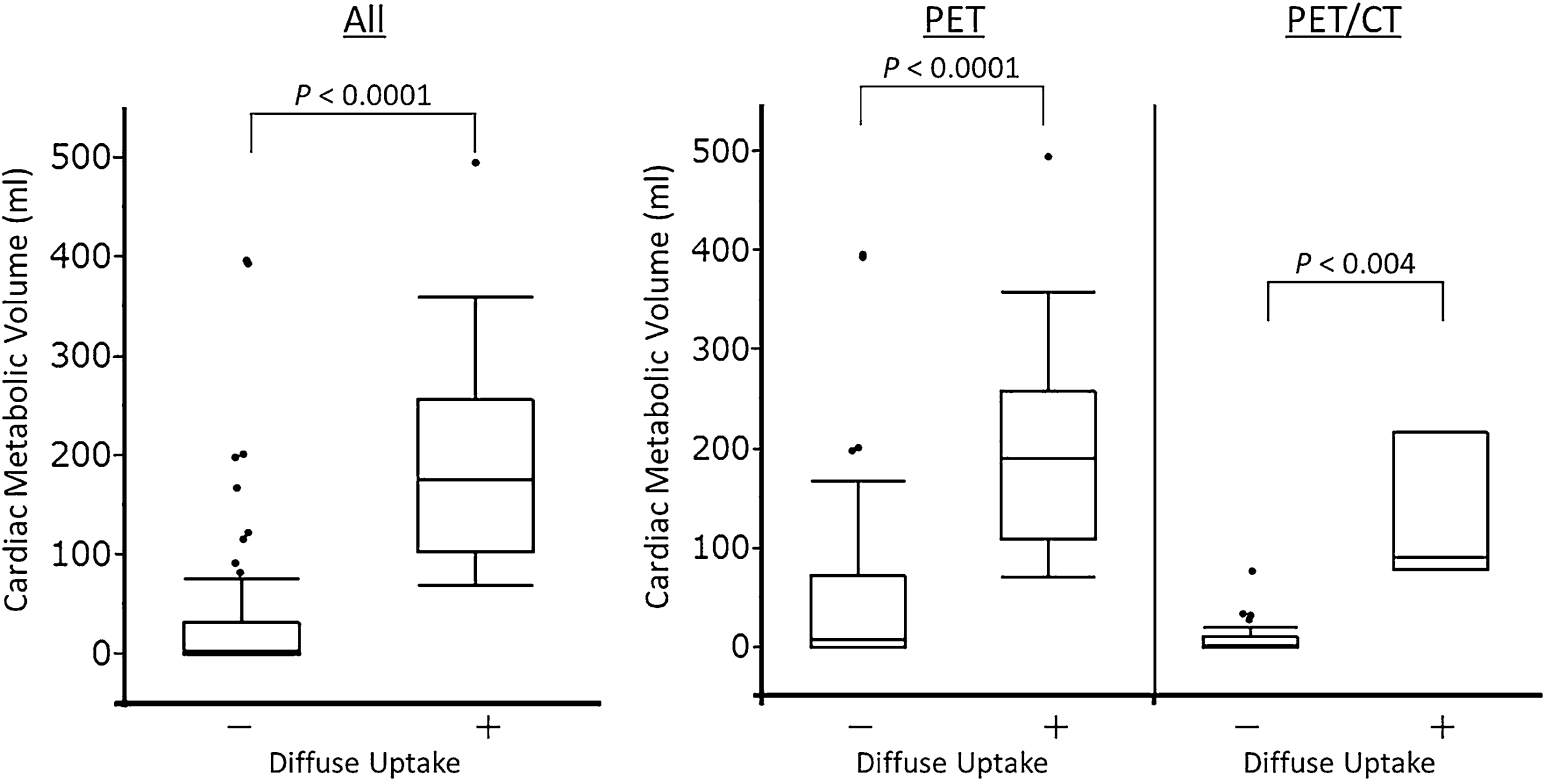
Comparison of cardiac metabolic volume. The cardiac metabolic volume in patients with diffuse uptake was significantly larger than it was in patients without diffuse uptake (194.6 ± 118.5 vs 35.9 ± 79.3 mL, *P* < .0001). The trend was similar for analysis by PET (211.1 ± 123.8 vs 56.1 ± 100.0 mL, *P* < .0001) or by PET/CT (128.8 ± 77.0 vs 9.2 ± 16.6 mL, *P* < .0001)

There were no discordant findings between the two observers. In group A, 16 patients (27.6%) showed diffuse LV uptake (Figure 2A). On the other hand, no patients (0.0%) in group B showed diffuse LV uptake (Figure 2B). Therefore, group A showed high frequencies of diffuse LV FDG uptake compared to group B (*P* = .0041) (Table 2). The mean ± SD values of the cardiac lesion and LV blood pool SUVmax were 1.9 ± 0.4 and 4.4 ± 2.1, for group A, and 2.0 ± 0.5 and 5.0 ± 3.1 for group B, respectively. There were no significant differences between group A and group B. There was also no significant correlation between FFA and SUVmax of the blood pool (*r* = 0.027, *P* = .84 for group A; *r* = 0.059, *P* = .78 for group B). The threshold of liver uptake during PET was significantly lower than it was for PET/CT (2.28 ± 0.53 vs 3.02 ± 0.64, *P* < .0001). The CMV was significantly larger in patients with diffuse uptake than in patients without diffuse uptake (PET: 211.1 ± 123.8 vs 56.1 ± 100.0 mL, *P* < .0001; PET/CT: 128.8 ± 77.0 vs 9.2 ± 16.6 mL, *P* = .004) (Figure 3).

**Table 2:** Prevalence of diffuse LV FDG uptake

	Group A	Group B
Diffuse LV FDG uptake (+)	16 (27.6%)*	0 (0.0%)
Diffuse LV FDG uptake (−)	42 (72.4%)	24 (100.0%)
Total	58	24

Group A: patients with minimum 6 h fasting without LCD preparation. Group B: patients with minimum 18 h fasting with LCD

*LV*, left ventricle; *LCD*, low-carbohydrate diet

*Group A showed high frequencies of diffuse LV FDG uptake when compared to group B (*P* = .0041)

### Blood Sample Data Before UFH Administration

There were no significant differences in the insulin levels of the two groups before UFH administration (Table 3). Group B showed lower FPG (84.3 ± 11.1 vs 93.3 ± 15.2 mg/dL, *P* = 0.001) and higher FFA (1159.1 ± 393.0 vs 650.5 ± 310.9 μEq/L, *P* < .0001) than did group A.

**Table 3:** Blood sample data

	Pre UFH injection			Post UFH injection	
	FPG (mg/dL)	IRI (μU/mL)	FFA (μEq/L)	IRI (μU/mL)	FFA (μEq/L)
Group A	93.3 ± 15.2	3.69 ± 2.93	650.5 ± 310.9	4.47 ± 2.38	2026.2 ± 712.1*
Group B	84.3 ± 11.1	3.16 ± 3.41	1159.1 ± 393.0	2.88 ± 3.67	2115.5 ± 531.3*
*P* value	.0010	.099	<.0001	<.0001	.52

Group A: patients with minimum 6 h fasting. Group B: patients with minimum 18 h fasting and LCD preparation

*UFH*, unfractionated heparin; *FPG*, fasting plasma glucose; *IRI*, immunoreactive insulin; *FFA*, free fatty acid; *LCD*, low-carbohydrate diet

*P < .0001 vs before UFH administration

**Figure 4:**
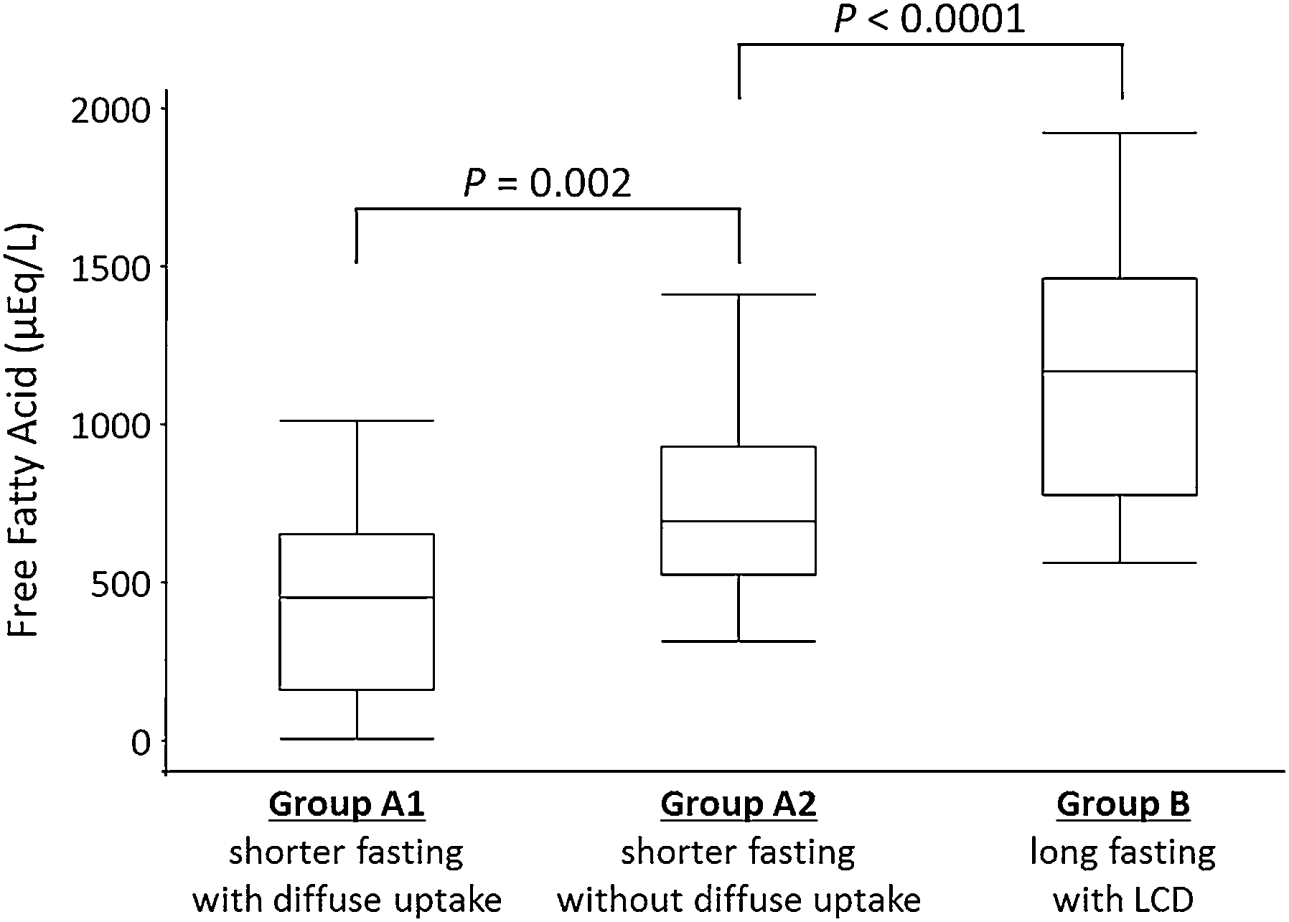
FFA levels for patients with and without diffuse LV FDG uptake. The group that fasted for longer and had a low-carbohydrate diet showed higher FFA levels before UFH injection than did the short-fast group. In the short-fast group, patients with LV diffuse FDG uptake showed significantly lower FFA levels than did patients without LV diffuse uptake before UFH injection. Group A1: patients with minimum 6 h fasting who showed positive diffuse LV FDG uptake. Group A2: patients with minimum 6 h fasting who showed negative diffuse LV FDG uptake. Group B: patients with minimum 18 h fasting and LCD preparation

In addition, in group A, patients of group A1 with diffuse LV FDG uptake showed significantly lower FFA levels than did patients in group A2 without diffuse LV FDG uptake before UFH injection (432.1 ± 296.1 vs 733.7 ± 276.8 μEq/L, *P* = .0023) (Figure 4).

### Blood Sample Data After UFH Administration

UFH administration significantly increased FFA in both groups (*P* < .0001). There were no significant differences in FFA levels for group A and those for group B 15 min after UFH injection (*P* = .52). In addition, with regard to FFA levels 15 min after administration of UFH, there was no significant difference between levels for group A1 and those for group A2 (1804.2 ± 739.7 vs 2110.8 ± 691.5 μEq/L, *P* = .26). Patients with diffuse LV FDG uptake (group A1) also had significantly increased plasma FFA after UFH (*P* < .0001) (Table 4).

**Table 4:** Blood sample data for patients with minimum 6 h of fasting

	Pre UFH injection			Post UFH injection	
	FPG (mg/dL)	IRI (μU/mL)	FFA (μEq/L)	IRI (μU/mL)	FFA (μEq/L)
Group A1	88.3 ± 6.0	4.04 ± 2.53	432.1 ± 296.1	4.46 ± 2.86	1804.2 ± 739.7*
Group A2	95.3 ± 17.2	3.55 ± 3.08	733.7 ± 276.8	4.47 ± 2.21	2110.8 ± 691.5*
*P* value	.052	.23	.0023	.60	.26

Group A1: patients who had positive diffuse LV FDG uptake. Group A2: patients who had negative diffuse LV FDG uptake

*UFH*, unfractionated heparin; *FPG*, fasting plasma glucose; *IRI*, immunoreactive insulin; *FFA*, free fatty acid; *LCD*, low-carbohydrate diet

*P < .0001 vs before UFH administration (group A1) and negative (group A2)

## Discussion

The minimum 18-h fasting with LCD preparation reduced FPG, increased FFA before UFH administration, and led to significantly suppressed diffuse LV FDG uptake, according to not only visual evaluation but also volume-based analysis, in comparison with results of shorter fasting without LCD protocol. UFH administration led to increases in FFA levels; however, the FFA levels after UFH administration might not play a significant role in reducing diffuse LV FDG uptake.

FDG is an analog of glucose and FDG PET is known to be useful for detecting inflammatory lesions. Sarcoidosis lesions include inflammatory cell infiltration by such things as lymphocytes and macrophages, which utilize glucose for their energy source. FDG PET can therefore be used to identify active sarcoidosis lesions.^2^ However, diffuse FDG uptake in the entire LV wall is generally considered to be physiologically normal.^1^ In the current study, the association between fasting conditions and diffuse LV FDG uptake was significant. Minimum 18-h fasting with LCD preparation led to significantly suppressed diffuse LV FDG uptake compared to results with the shorter fasting protocol. These data may imply the importance of a gradual myocardial metabolic shift from glucose use to fatty acid use in fasting FDG imaging. The myocardium could derive energy from a variety of sources such as FFA, glucose, lactate, and ketone bodies.^11^ Several factors were known to affect the utilization of an individual substrate, including the plasma insulin concentration, availability of alternative substrates, and myocardial blood flow supply.^25^ The heart preferentially utilizes substrates that yield energy from FFA and lactate, while glycolysis normally produces only about 30% of substrates for the citric acid cycle.^26^ In particular, the heart prefers to use FFA for energy production, and under these conditions, myocardial glucose uptake is low with fasting.^15^ In the absence of dietary glucose intake and carbohydrate intake due to prolonged fasting, the myocardium physiologically adapts and makes a metabolic shift from glucose use to fatty acid use. Under the long fasting condition, glucose production and glucose oxidation should be decreased. In contrast, FFA is mobilized from adipose tissue and the increased FFA becomes an alternative fuel in the myocardium.^27^ Wisneski et al demonstrated the presence of a significant inverse correlation between the arterial FFA level and myocardial glucose uptake in normal healthy subjects.^28^ Specifically, after a carbohydrate meal, FFA uptake and oxidation decrease, and glucose and lactate extraction increase.^29^ It has been suggested that this shift in fuel preference is caused by the changes in substrate and leads to the suppression of physiological glucose metabolism. In fact, the current data showed higher plasma FFA levels in the group of patients undergoing long fasting with LCD. This increasing FFA use may be significantly reduced by cardiac glucose metabolism. Thus, long fasting with LCD preparation significantly reduces physiological FDG uptake. In addition, even with shorter fasting, some patients in the current study showed significantly reduced physiological myocardial FDG uptake. These patients also showed significantly higher plasma FFA levels than did patients with diffuse LV FDG uptake. This result may indicate the importance of increasing plasma FFA levels for fasting cardiac FDG PET imaging or of measuring plasma FFA before the fasting FDG PET study.

Langah et al reported the effects of 18-h fasting preparation on the suppression of myocardial physiological FDG uptake.^13^ They indicated that the frequency of LV FDG uptake under prolonged fasting was significantly lower than under the short fasting protocol. In their report, only one of the 10 patients (10.0%) had a diffuse pattern. Morooka et al also reported the importance of prolonged fasting for evaluating active inflammatory lesions in cardiac sarcoidosis.^30^ In their study, healthy volunteers who fasted for 18 h showed reduced physiological LV FDG uptake in comparison with healthy volunteers who underwent 12 h of fasting with heparin administration. In addition, they suggested that the FFA level before UFH injection may be the important factor in inhibiting physiological LV FDG uptake. Physiological uptake was likely to be efficiently inhibited when the FFA level was more than 760 μEq/L. Our current data agree with their previous study. In this study, we applied the longer fasting protocol to CS patients and showed the usefulness of this preparation for eliminating diffuse LV FDG uptake in clinical settings. Thus, the current study added further insights to those of previous studies by Langah et al^13^ and Morooka et al.^30^

In the current study, the 18-h fasting group also followed LCD. LCD also has an impact on myocardial metabolism and may contribute to reducing physiological FDG uptake.^31^ Kobayashi et al reported the usefulness of LCD in suppressing myocardial physiological FDG uptake in healthy subjects.^22^ Kumar et al reported that both restricted diet and duration of fasting play an important role in determining the pattern and suppression of myocardial FDG uptake.^32^ LCD may have played a role in reducing the physiological LV FDG uptake in the present study; however, since we did not apply LCD to the shorter fasting group, we are not able to conclude which factor played a more significant role in the current study. Further study examining the effects of LCD on myocardial physiological FDG uptake under various fasting conditions should be conducted in the future.

UFH activates lipoprotein lipase, enhances plasma lipolytic activity, and increases FFA levels.^33^,^34^ Therefore, UFH has been expected to reduce physiological LV FDG uptake. In this regard, our group applied UFH for fasting cardiac FDG PET studies in CS.^14^,^21^ However, the full effects of heparin in reducing physiological LV FDG uptake have not been established.^1^ In the current study, UFH increased plasma FFA levels in both group A and group B. In addition, in the short-fast group, the difference in FFA levels after 15 min of UFH administration between patients with diffuse LV uptake and those without diffuse LV uptake was not significant. The current data agree with those of previous reports indicating that patients with increased FFA responded to UFH.^14^,^35^ Moreover, the present data may imply that acutely increasing plasma FFA by UFH may not have a significant role in reducing physiological LV FDG uptake. A recent study by Gormsen et al reported that heparin loading did not reduce myocardial FDG uptake.^36^ Our data agree with previous data and also may imply the importance of longer fasting with LCD rather than heparin administration. However, the effects of heparin should also be evaluated in future studies.

Distribution of FDG uptake might change in patients with diabetes who use insulin or oral antidiabetic drugs.^37^ There were four diabetic patients in this study. Two of them were in group A and the other two were in group B. Their FPG levels were lower than 114 mg/dL, and their uptake patterns were not diffuse. If their results were excluded, the conclusion remained the same.

## Limitations

The present study had some limitations. We used both PET and PET/CT systems. The detector and method of attenuation correction were not same. However, the current visual analyses focused on the existence of diffuse physiological myocardial FDG uptake. This visual approach was simple and relatively independent from attenuation correction or other system specifications. A previous study by Youssef et al assessed cardiac involvement sarcoidosis using 3 different PET scanners from 3 different centers.^4^ In the quantitative analysis, each system showed that the cardiac metabolic volume was significantly larger in the diffuse uptake group. The results were consistent. Thus, the effects of using different PET systems may have minimal impact on the current study similar to the previous study by Youssef et al.

Patients in group A were instructed to fast for a minimum of 6 h; however, the actual fasting time of group A was not available. For the patients in group B, fasting time was based on self-reporting. However, all patients were admitted to hospital and were provided dinner at the same time, and therefore, reported fasting times should be accurate. Obvious whole LV FDG accumulation higher than liver uptake was defined as diffuse LV FDG uptake. However, physiological uptake may sometimes be seen in only part of the LV myocardium rather than in the whole, and therefore, further investigation to reveal the relationship between other patterns of FDG uptake and fasting conditions is warranted.

Recently, prolonged fasting and diet modifications including a low-carbohydrate diet and a high-fat diet have been applied to FDG PET/CT studies in patients with CS. In the current study, we did not evaluate the effects of high-fat meals. The effects of adding a high-fat diet should be studied as the next step.

## New Knowledge Gained

The minimum 18-h fasting with LCD preparation reduced FPG, increased FFA before UFH administration, and led to significantly suppressed diffuse LV FDG uptake in comparison with results of shorter fasting without LCD protocol. In particular, the FFA level before UFH injection was significantly lower in patients with LV diffuse uptake than in patients without LV diffuse uptake. After the UFH administration, both groups had significantly increased plasma FFA. However, the shorter fasting group had a higher frequency of physiological FDG uptake. Therefore, UFH might not play a significant role in reducing diffuse LV FDG uptake. This finding adds new insight over that from previous studies.

## Conclusions

Fasting for more than 18 h combined with a low-carbohydrate diet preparation significantly reduced physiological LV FDG uptake and increased FFA levels prior to UFH administration in comparison with the case for shorter fasting preparations. There were no significant differences in FFA levels after UFH injection between patients with diffuse LV FDG uptake and patients without diffuse LV FDG uptake. Therefore, this protocol of prolonged fasting with a low-carbohydrate diet preparation may be useful for detecting inflammatory regions of CS in clinical settings.

## Abbreviations

CAD: Coronary artery disease
CS: Cardiac involvement sarcoidosis
FDG: ^18^F-fluorodeoxyglucose
FFA: Free fatty acid
FPG: Fasting plasma glucose
JMHW: Japanese Ministry of Health and Welfare
LCD: Low-carbohydrate diet
LV: Left ventricle
UFH: Unfractionated heparin
